# The Role of CCTA-derived Cardiac Structure and Function Analysis in the Prediction of Readmission in Nonischemic Heart Failure

**DOI:** 10.1007/s12265-023-10467-6

**Published:** 2024-01-26

**Authors:** Chengjia Liu, Shuangxiang Lin, Yangyang Sheng, Xinghong Wang, Jianzhong Sun, Jiaxing Wu, Risheng Yu

**Affiliations:** 1 Department of Radiology, The Second Affiliated Hospital Zhejiang University School of Medicine, Hangzhou, Zhejiang China; 2 Siemens Healthineers, No.399, West Haiyang Road, Shanghai, 200126 China

**Keywords:** Nonischemic heart failure, Coronary CT angiography, Epicardial adipose tissue, Peri-coronary adipose, Fractional flow reserve

## Abstract

Cardiac function and structure significantly impact nonischemic heart failure (HF) patient outcomes. This study investigated 236 patients (107 nonischemic heart failure, 129 healthy) to assess the relationship between coronary computed tomography angiography (CCTA)-derived parameters and clinical outcomes. Among the nonischemic heart failure patients, 37.3% experienced readmissions. In this group, specific CCTA measurements were identified as significant predictors of readmission: epicardial adipose tissue (CT_EAT_) at 54.49 cm^3^ (HR: 1.05; 95% CI: 1.03–1.07; *P* < 0.001), cardiac muscle mass to lumen volume (CT_V/M_) at 20% (HR: 0.59; 95% CI: 0.48–0.72; *P* < 0.001), peri-coronary adipose (CT_PCAT_) at -64.68 HU (HR: 1.1; 95% CI: 1.03–1.16; *P* = 0.002) for the right coronary artery, -81.07 HU (HR: 1.3; 95% CI: 1.1–1.53; *P* = 0.002) for the left anterior descending artery, and -73.42 HU (HR: 1.33; 95% CI: 1.18–1.51; *P* < 0.001) for the circumflex branch of the left coronary artery. In patients with nonischemic heart failure, increased CT_EAT_, CT_PCAT_, and CT_V/M_ independently predicted rehospitalization.

**Graphical Abstract:**

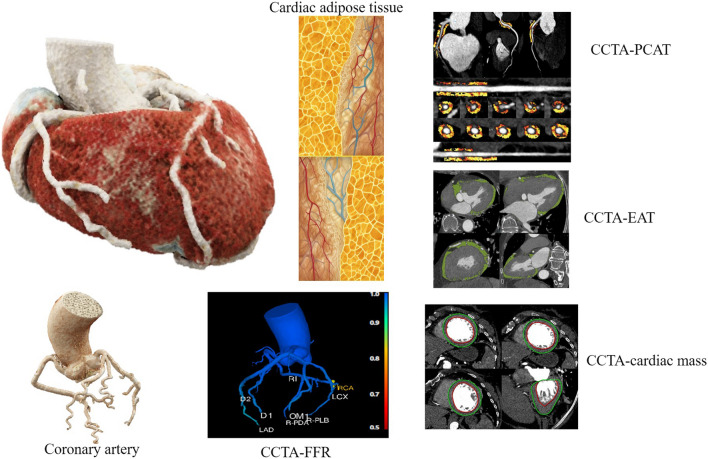

**Supplementary Information:**

The online version contains supplementary material available at 10.1007/s12265-023-10467-6.

## Introduction

Patients with heart failure (HF) experience mortality and hospital readmission rates exceeding 15% and 30%, respectively, within 60 to 90 days post-discharge, placing a significant burden on medical systems [1]. HF can be classified into ischemic and nonischemic types according to the 2022 AHA/ACC/HF-SA Guidelines, both of which pose, complex challenges in clinical practice [2, 3]. Nonischemic HF (non-ICHF), characterized by myocardial hypertrophy, valve dysfunction, myocardial inflammation, and various structural and functional abnormalities, lacks effective prognostic biomarkers [4]. While biomarkers such as high-sensitivity C-reactive protein (hs-CRP), B-type natriuretic peptide (BNP), N-terminal pro-brain natriuretic peptide (NT-proBNP), and left ventricular ejection fraction (LVEF) have been broadly applied in assessing the prognosis of non-ICHFHF patients, their limitations in providing accurate prognostic information persist [5, 6].

The application of computed tomography coronary angiography (CCTA) in non-ICHF has emerged as a burgeoning focus within the field of cardiovascular medicine [7]. While initially primarily utilized for the assessment of coronary artery diseases, CCTA has expanded its scope to encompass comprehensive evaluations of cardiac structure and function, offering novel diagnostic and therapeutic opportunities for non-ICHF patients [8, 9]. First, CCTA employs high-resolution, three-dimensional imaging, which can be used to depict the anatomical structure of coronary arteries, facilitating the exclusion of coronary artery stenosis or obstruction [10]. Second, CCTA is employed for the assessment of cardiac structure, including myocardial thickness, ventricular wall motion abnormalities, valvular function, and the calculation of the cardiac lumen volume to muscle mass (V/M) ratio [6, 11], all of which are pivotal factors in the development and treatment of non-ICHF. Recent research has also indicated the potential of CCTA to measure epicardial adipose tissue (EAT) and pericoronary adipose tissue (PCTA), factors of emerging significance in non-ICHF investigations [12–14]. Therefore, CCTA provides non-ICHF patients with a multifaceted, noninvasive assessment option, integrating information from coronary arteries, cardiac structure and function, and cardiac adipose tissue. It holds potential as a potent tool for prognosis assessment and treatment decision-making, offering new hope for the management and prognosis of non-ICHF patients.

In this research, we utilize EAT and the V/M ratio to assess cardiac metabolic capacity, PCAT to assess cardiac inflammatory responses, and the CT-derived fractional flow reserve (CT_FFR_) to evaluate coronary hemodynamics. Our objective was to construct an integrated model for prognosticating the clinical outcomes of these patients.

## Methods

### Study Population

**Fig. 1:**
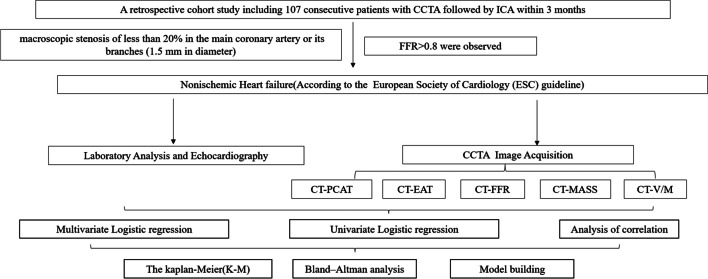
Study flow chart

Between January 2019 and June 2022, a retrospective cohort study including 107 consecutive patients who underwent CCTA followed by invasive coronary angiography (ICA) within 3 months at The Second Affiliated Hospital, Zhejiang University School of Medicine, Hangzhou, Zhejiang, China, was performed. A total of 129 healthy controls were also included in the study to compare the CCTA-derived parameters of the patients with those in the normal population. A flowchart of this study is illustrated in Fig. 1.

All participants were of Asian ethnicity. The inclusion criteria were as follows: (1) heart failure defined according to the European Society of Cardiology (ESC) guidelines [2]; (2) New York Heart Association [NYHA] class II-IV or signs consistent with the Framingham criteria; (3) no coronary artery stenosis on coronary angiography; and. (4) CT-FFR > 0.8. The exclusion criteria included (1) missing images or poor image quality; (2) incomplete laboratory tests; and (3)) incomplete ultrasound data and loss to follow-up. All patient information, including demographic characteristics, medical history, laboratory tests, and echocardiography results, was collected after admission.

All patients provided informed consent. This study was approved by the Human Research Ethics Committee of the Second Affiliated Hospital, Zhejiang University School of Medicine (I20221200).

### Data collection

Medical records including medical history, laboratory data, medication, and clinical course were reviewed. Laboratory blood analysis was performed within 48 h before CCTA. Based on previous literature, metabolic syndrome was diagnosed in patients with a body-mass index (BMI) greater than 25 kg/m^2^ and two or three of the following conditions [15]: (1) fasting plasma glucose ≥ 110 mg/dl or HbA1c (NSGP) ≥ 5.5%, (2) systolic blood pressure ≥ 130 mmHg or diastolic blood pressure ≥ 85 mmHg, and (3) triglycerides ≥ 150 mg/dl or high-density lipoprotein (HDL) cholesterol < 40 mg/dl. Throughout the entire echocardiographic assessment, a single-lead electrocardiogram was recorded. Following the guidelines of the American Society of Echocardiography, a phased-array transducer with a fusion frequency of 2–4 MHz was employed [16]. Imaging was conducted in M-mode and 2D modes, capturing images in parasternal long-axis, short-axis, apical four-chamber, and two-chamber views and at the aortic root for evaluation. Measurements included the thicknesses of the interventricular septum (IVS), the anterior wall of the left ventricle (LVID), and the posterior wall of the left ventricle (LVPW) during both systole and diastole of the heart. The diameter of the connection point between the aorta and the sinus of Valsalva (AO-stj) and anteroposterior diameter of the left atrium (LA-ap) were also measured. LVEF was calculated using the biplane Simpson's method, averaged over three consecutive heartbeats. BMI was calculated as weight (kilograms) divided by height (meters) squared.

### Cardiac Computed Tomography Protocol and Image Acquisition

All participants underwent dual-source CT scans (Somatom Definition Flash or SOMATOM Force, Siemens Healthcare, Germany), which included coronary artery calcium scoring (CACS) and CCTA. The following parameters were used for the CACS protocol: tube voltage, 120 kV; tube current, 80 mAs with automated tube current modulation (CARE Dose 4D, Siemens Healthineers); and section thickness, 2 mm with a 1.5 mm increment. CCTA was conducted with a prospective ECG-gated sequence: CARE kV (reference tube voltage, 100 kV);); CARE Dose 4D (reference tube current, 288 mAs); acquisition phase, 30–75%; 65% R-R interval; and reconstructed slice thickness, 0.75 mm with a 0.5 mm increment. The images were reconstructed by advanced model iterative reconstruction (ADMIRE) level 3; kernel Bv40. Contrast medium was administered at a dose ranging from 30 to 60 mL, and the flow rate ranged from 4.0 to 6.0 mL/s according to the patients’ BMI, heart rate, and tube voltage. CCTA was performed after the injection of contrast medium (iodine 370 mg/mL [Ultravist, Bayer Schering Pharma, Berlin, Germany]) via an 18–20 G intravenous catheter, followed by a 30 mL saline flush using a dual-head power injector.

### CT-derived and structural function index measurement

The CT-derived EAT (CT_EAT_), mass (CT_MASS_), V/M (CT_V/M_), FFR (CT_FFR_) and PCAT (CT_PCAT_) were assessed on a workstation (syngo.via VB40, Siemens Healthineers, Germany). Two radiologists who were blinded to the patient data (SX Lin [observer 1] and CJ Liu [observer 2], with over 5 years of experience in CT diagnostic imaging). CT_FFR_ was calculated and analysed based on a software prototype (cFFR, syngo.via Frontier, version 3.0.1, Siemens Healthineers, Germany).

CT_EAT_, defined as the total amount of adipose tissue between the surface of the heart and the visceral layer of the pericardium, was measured by volumetry on short-axis slices with a thickness of 0.75 mm, ranging from the level of the pulmonary bifurcation to the apex and within a threshold range of -190 to -30 Hounsfield units (HU) to determine the total volume of tissue [17]. The adipose tissue around the coronary artery is part of the EAT and was defined as the attenuation coefficient of the fat tissue voxels within a distance from the coronary arterial wall equal to the corresponding vessel diameter. The attenuation coefficient was calculated separately for the right coronary artery (RCA), left anterior descending artery (LAD), and left circumflex artery (LCX) using the coronary artery analysis module [18].

The cardiac mass was determined using the syngo.via cardiac analysis module, which automatically traces the endocardial and epicardial borders to generate the volume of the heart. The myocardial volumes were converted to left ventricle mass (M) by assuming a constant tissue density of myocardium (1.05 g/cc). CT_V/M_ was calculated by dividing the luminal volume of the coronary artery by the myocardial mass of the heart [9, 19]. The luminal volume of the coronary artery was extracted by a deep learning calculation method on the workstation. In this study, the right coronary artery was selected for coronary lumen volume analysis given its simplicity and thick diameter in the Chinese population.

### Follow-up

Follow-up included follow-up data and results obtained from January 2019 and June 2022 in the HIS system as a follow-up of disease and care, clinic visits, and telephone interviews. The endpoints were patient readmission (defined as a hospital admission for which HF was the primary reason and requiring either diuretic, inotropic, or intravenous nitrate therapy). All statuses were reviewed by 2 independent investigators who used previously described criteria.

### Statistical Analysis

Data were analysed using R software (Version 4.0). Continuous variables are reported as the mean ± SD or median (interquartile range, IQR) and were compared using independent samples t tests and one-way analysis of variance or Wilcoxon signed-rank and Kruskal‒Wallis tests. Categorical variables are presented as absolute values and percentages. The correlation between healthy controls and HF patients was analysed using the Pearson correlation. Inter- and intrao bserver agreement for the CT_EAT_, CT_MASS_, CT_V/M_, CT_FFR_ and CT_PCAT_ were performed using Bland–Altman analysis.

For survival analysis, survival and proportional hazards assumptions were estimated by the Kaplan‒Meier method, and any differences were evaluated with the stratified log-rank test. The optimal cut-off point was identified using the maximally selected rank statistic (maxstat) [20], which is a ranking statistic for maximum selection. Odds ratios (ORs) and 95% confidence intervals (CIs) were determined from univariate and multivariate logistic regression analyses to determine any factors associated with the endpoint. A favourable patient prognosis was taken as the dependent variable, and the statistically significant factors were included in the logistic regression model as independent variables for regression analysis. Three risk models were created based on the best traditional parameter variables (model 1), the CCTA-driven parameters (model 2), and the combination of the two sets of parameters (model 3). Receiver operating characteristic (ROC) curve analysis was performed to evaluate the prognostic accuracy of CT_EAT_, CT_MASS_, CT_V/M_, CT_FFR_ and CT_PCT_ for the endpoint.

## Results

### Baseline Patient Characteristics

The clinicopathological characteristics of the patients are summarized in Table 1. A total of 236 patients were enrolled in this clinical study. Compared to the healthy control group, the non-ICHF group was significantly different in age, height, diastolic blood pressure and metabolic syndrome. Among the laboratory data, CHO tended to be lower in the non-ICHF than in the control group (3.9 mmol/L vs. 3.6 mmol/L, *P* = 0.003), while TG (1.3 mmol/L vs. 1.1 mmol/L, *P* = 0.009), HDL-C (1.2 mmol/L vs. 1.1 mmol/L, *P* = 0.004), and LDL_C (1.9 mmol/L vs. 1.7 mmol/L, *P* = 0.0035) were significantly different (Table 1).

**Table 1:** Demographic characteristics of the healthy control and non-ischemic HF

	[ALL]	Control	Non-ICHF	*P*-Value
	*N* = 236	*N* = 129	*N* = 107	
Clinical history				
Gander				0.686
Female	97 (41.1%)	51 (39.5%)	46 (43.0%)	
Male	139 (58.9%)	78 (60.5%)	61 (57.0%)	
Age	68.1 (9.9)	65.0 (9.3)	71.8 (9.4)	< 0.001*
Weight (kg)	63.3 [58.0;70.2]	65.0 [58.0;72.8]	62.7 [58.0;67.0]	0.133
Height (cm)	165.0 [158.0;170.0]	165.0 [160.0;172.0]	162.0 [155.0;170.0]	0.007*
Body mass index (kg/m2)	23.14 ± 3.6	23.8 ± 2.8	24.89 ± 3.1	0.005*
Systolic (mmHg)	133.5 (18.8)	132.3 (19.0)	134.9 (18.6)	0.284
Diastolic (mmHg)	73.3 (11.4)	74.9 (10.6)	71.5 (12.0)	0.022*
Hypercholesterolemia	28 (11.9%)	15 (11.7%)	13 (12.1%)	1
Hypertension:	148 (62.7%)	77 (59.7%)	71 (66.4%)	0.358
Diabetes:	61 (25.8%)	28 (21.7%)	33 (30.8%)	0.148
Smoking:	85 (36.2%)	43 (33.6%)	42 (39.3%)	0.446
CAD history	21 (8.9%)	9 (7.0%)	12 (11.2%)	0.373
Metabolic syndrome	68 (61.6%)	12 (9.3%)	56 (52.3%)	0.002*
Laboratory data				
CHO (mmol/L)	3.8 [3.3;4.5]	3.9 [3.4;4.5]	3.6 [3.1;4.2]	0.003*
TG (mmol/L)	1.2 [0.9;1.6]	1.3 [1.0;1.8]	1.1 [0.8;1.5]	0.009*
HDL-C (mmol/L)	1.2 [1.0;1.4]	1.2 [1.0;1.4]	1.1 [0.9;1.3]	0.004*
LDL-C (mmol/L)	1.8 [1.5;2.4]	1.9 [1.6;2.4]	1.7 [1.4;2.1]	0.035*
ApoA1 (g/L)	1.4 [1.3;1.5]	1.4 [1.3;1.6]	1.4 [1.3;1.5]	0.12
ApoB (g/L)	0.7 [0.6;0.8]	0.7 [0.6;0.8]	0.7 [0.6;0.8]	0.918
CRP (mg/L)	3.5 [1.7;7.8]	3.1 [1.5;6.6]	4.0 [1.9;10.4]	0.054
FFA (mmol/L)	317.1 [210.8;457.0]	301.4 [204.4;470.7]	320.2 [220.8;436.7]	0.646
CCTA variables				
EAT (cm^3^)	47.3 [38.2;56.3]	39.6 [31.3;47.6]	55.0 [48.9;64.2]	< 0.001*
PCAT_RCA_ (HU)	-67.7 [-64.0;-72.4]	-67.0 [-62.5;-71.4]	-68.9 [-65.3;-73.1]	0.004*
PCAT_LAD_ (HU)	-78.4 [-76.2;79.9]	-77.2 [75.4;78.8]	-79.8 [-78.2;-81.3]	< 0.001*
PCAT_LCX_ (HU)	-69.6 [-65.4;-73.3]	-68.2 [-64.2;-70.7]	-73.3 [-67.1;-75.4]	< 0.001*
CT_FFR_	0.9 [0.9;1.0]	1.0 [0.9;1.0]	0.9 [0.8;0.9]	< 0.001*
V (cm)	19.9 ± 2.3	21.1 ± 2.0	18.5 ± 1.6	< 0.001*
M (g)	81.0 [76.3;87.0]	81.0 [76.0;85.0]	82.0 [76.7;89.0]	0.036*
V/M (%)	24.1 [23.0;26.5]	26.2 [24.4;27.8]	22.0 [21.0;24.0]	< 0.001*

Data are presented as mean ± SD, median (IQR) or % (n).BMI body mass index; CHO cholesterol; TG triglyceride, HDL-C High density liptein cholesterol, LDL_C Low density liptein cholesterol, ApoA1 Apolipoprotein A1, ApoB apolipoprotein B, FFA free fatty acid; BNP, B-type natriuretic peptide, NT-proBNP pro-B type natriuretic peptide, CT_EAT_ CT derived epicardial adipose tissue; CT_PCAT_ CT derived peri-coronary adipose, CT_V/M_ CT derived cardiac muscle mass to lumen volume, CT_FFR_ CT derived fractional flow reserve;

^*^ Significant difference

### CCTA-Derived Parameters

**Fig. 2:**
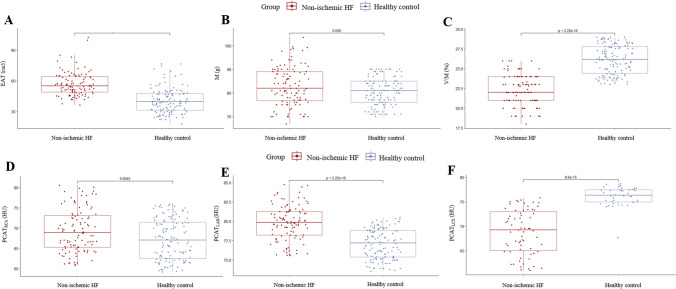
The Boxplot for CCTA-derived parameters in healthy control and non-ischemic Heart failure; (**A**) CT derived epicardial adipose tissue; (**B**) CT derived cardiac muscle mass; (**C**) CT derived cardiac muscle mass to lumen volume; (**D**) peri-coronary adipose in the right coronary artery; (**E**) peri-coronary adipose in the left anterior descending artery; (**F**) peri-coronary adipose in the circumflex branch of the left coronary artery

Except for CT_V/M_ (26.2% [IQR, 24.4; 27.8] vs. 22.0% [IQR, 21.0; 24.0], *P* < 0.01), the CCTA-derived parameters in the healthy groups were significantly lower (*P* < 0.05) than those in the non-ICHF group (Table 1). In non-ICHF, the volume of adipose tissue around the proximal LAD (-79.8 HU [IQR, -78.2; -81.3]) was higher than that around the RCA (-68.9 HU [IQR, -65.3; -73.1]) and LCX (-73.3 HU [IQR, -67.1; -75.4]) (Table 1, Fig. 2).

There was a moderate to strong correlation between healthy patients and non-ICHFHF patients in the attenuation of the PCAT of the LAD (r = 0.55; P < 0.0001), the right circumflex artery and LCX (r = 0.422; P < 0.0001), the coronary lumen volume (r = -0.57; P < 0.0001) and CT-derived cardiac V/M ratio (r = -0.72; P < 0.0001) (Supplementary Fig. 1).

### Clinical Endpoints

The last follow-up was in October 2022. During a mean follow-up of 6 ± 0.9 months (range 2–28 months), 40 patients were rehospitalized for heart failure. These patients demonstrated a significant difference in terms of HDL-C (1.1 mmol/L [0.9;1.2] vs. 1.2 mmol/L [1.1;1.4], *P* = 0.005) and CRP (4.8 mg/L [2.5;14.1] vs. 3.5 mg/L [1.5;7.0], *P* = 0.033) (Table 2). Univariate logistic regression analysis showed that HDL-C (OR: 7.68, 95% CI: 1.57–3.76, *P* = 0.08) and CRP (OR: 0.92, 95% CI: 0.85–0.99, *P* = 0.08) were associated with readmission. The multivariable regression model showed that HDL-C (OR: 2.35, 95% CI: 1.31–4.29, *P* = 0.08) and TG (OR: 2.16,: 1.14–3.12, *P* < 0.01) were significantly associated with readmission in the study population (Table 3).

**Table 2:** Demographic characteristics of the readmission non-ischemic HF

	[ALL]	No event	Event	*P*-Value
	*N* = 107	*N* = 67	*N* = 40	
Gander(M)	61 (57.0%)	36 (53.7%)	25 (62.5%)	0.494
Age	71.8 (9.4)	70.9 (9.3)	73.4 (9.6)	0.194
Weight (kg)	62.7 [58.0;67.0]	63.0 [58.2;67.0]	61.8 [58.0;68.2]	0.99
Hight (cm)	162.0 [155.0;170.0]	161.0 [154.7;167.5]	165.0 [157.2;170.0]	0.116
Body mass index (kg/m2)	23.14 ± 3.6	23.8 ± 2.8	24.89 ± 3.1	
systolic(mmHg)	134.9 (18.6)	136.8 (18.0)	131.8 (19.5)	0.195
diastolic(mmHg)	71.4 [64.0;78.0]	72.0 [65.5;78.0]	69.0 [61.8;78.0]	0.491
Hypercholesterolemia:	13 (12.1%)	9 (13.4%)	4 (10.0%)	0.763
Hypertension:	71 (66.4%)	45 (67.2%)	26 (65.0%)	0.986
Diabetes:	33 (30.8%)	25 (37.3%)	8 (20.0%)	0.097
Smoking:	42 (39.3%)	27 (40.3%)	15 (37.5%)	0.934
CAD history	12 (11.2%)	10 (14.9%)	2 (5.0%)	0.204
Metabolic syndrome	56 (52.3%)	35 (52.2%)	21 (52.5%)	0.364
statins:	94 (87.9%)	55 (82.1%)	39 (97.5%)	0.029*
Aspirin:	90 (84.1%)	54 (80.6%)	36 (90.0%)	0.311
Clopidogrel:	52 (48.6%)	36 (53.7%)	16 (40.0%)	0.24
Bisoprolol:	6 (5.6%)	6 (9.0%)	0 (0.0%)	0.082
metoprolol:	37 (34.6%)	24 (35.8%)	13 (32.5%)	0.889
Metformin:	17 (15.9%)	14 (20.9%)	3 (7.5%)	0.119
Warfarin:	**9 (8.4%)**	**9 (13.4%)**	**0 (0.0%)**	**0.025**
CHO (mmol/L)	3.6 [3.1;4.2]	3.5 [3.2;4.1]	3.6 [3.0;4.4]	0.669
TG (mmol/L)	1.1 [0.8;1.5]	1.1 [0.8;1.6]	1.0 [0.7;1.5]	0.196
HDL_C (mmol/L)	**1.1 [0.9;1.3]**	**1.1 [0.9;1.2]**	**1.2 [1.1;1.4]**	0.005*
LDL_C (mmol/L)	1.7 [1.4;2.1]	1.8 [1.5;2.0]	1.7 [1.3;2.3]	0.903
ApoA1 (g/L)	1.4 [1.3;1.5]	1.4 [1.3;1.5]	1.4 [1.3;1.5]	0.88
ApoB (g/L)	0.7 [0.6;0.8]	0.7 [0.6;0.8]	0.7 [0.7;0.8]	0.434
CRP (mg/L)	**4.0 [1.9;10.4]**	**4.8 [2.5;14.1]**	**3.5 [1.5;7.0]**	0.033*
FFA (mmol/L)	320.2 [220.8;436.7]	316.2 [223.2;413.0]	345.6 [218.0;492.8]	0.352
EF (%)	0.7 [0.6;0.7]	0.7 [0.6;0.7]	0.7 [0.6;0.7]	0.404
IVSd (mm)	1.0 [0.9;1.0]	1.0 [0.9;1.0]	1.0 [0.9;1.1]	0.661
LVIDd (mm)	4.5 (0.5)	4.5 (0.5)	4.6 (0.5)	0.682
LVPWd (mm)	1.0 [0.9;1.0]	1.0 [0.9;1.0]	1.0 [0.9;1.0]	0.792
IVSs (mm)	1.4 [1.3;1.5]	1.4 [1.3;1.5]	1.4 [1.2;1.5]	0.139
LVIDs (mm)	2.9 [2.6;3.1]	2.9 [2.7;3.1]	2.9 [2.6;3.3]	0.65
LVPWs	1.4 [1.3;1.5]	1.4 [1.3;1.5]	1.4 [1.3;1.5]	0.804
AO.STJ	2.9 [2.7;3.1]	2.9 [2.7;3.1]	2.9 [2.7;3.0]	0.507
LA.ap	3.8 (0.5)	3.7 (0.5)	3.9 (0.4)	0.125
EAT (cm^3^)	**55.0 [48.9;64.2]**	**51.7 [47.1;57.2]**	**63.9 [55.5;69.5]**	< 0.001*
PCAT_RCA_ (HU)	**-68.9 [-65.3;-73.1]**	**-66.5 [-64.1;-71.1]**	**-71.2 [-67.8;-77.6]**	< 0.001*
PCAT_LAD_ (HU)	**-79.8 (2.2)**	**-79.0 (2.0)**	**-81.1 (2.0)**	< 0.001*
PCAT_LCX_ (HU)	**-73.3 [-67.1;-75.4]**	**- 69.2 [-65.1;-73.0]**	**-76.3 [-75.0;-77.4]**	< 0.001*
CT_FFR_	0.9 [0.8;0.9]	0.9 [0.8;0.9]	0.9 [0.8;0.9]	0.987
V(cm)	18.5 (1.6)	18.6 (1.6)	18.3 (1.6)	0.335
M(g)	82.9 (8.2)	79.4 (6.4)	88.6 (7.5)	< 0.001*
V/M (%)	**22.0 [21.0;24.0]**	**23.0 [22.2;24.0]**	**21.0 [20.0;21.0]**	< 0.001*

Data are presented as mean ± SD, median (IQR) or % (n).BMI Body Mass Index; CHO cholesterol; TG triglyceride, HDL-C High density liptein cholesterol, LDL_C Low density liptein cholesterol, ApoA1 Apolipoprotein A1, ApoB apolipoprotein B, FFA free fatty acid; BNP, B-type natriuretic peptide, NT-proBNP pro-B type natriuretic peptide, CTEAT CT derived epicardial adipose tissue; CTPCAT CT derived peri-coronary adipose, CTV/M CT derived cardiac muscle mass to lumen volume, CTFFR CT derived fractional flow reserve;

^*^ Significant difference

**Table 3:** Univariable and Multivariable logistic Analysis for the Prediction of non-ischemic HF readmission

Variable	Univariate Logistic			Multivariate Logistic		
	Regression analysis			Regression analysis		
	OR	95%CI	*P*-value	OR	95%CI	*P*-value
TG	0.53	0.25–1.13	0.05	-	-	-
HDL_C	**7.68**	**1.57–3.76**	**0.008**	**2.35**	**1.31–4.29**	**0.004**
CRP	**0.92**	**0.85–0.99**	**0.008**	-	-	-
TG	0.53	0.25–1.13	0.05	**2.16**	**1.14–3.12**	** < 0.001**
EF	20.2	0.55–1.9	0.069	-	-	-
EAT	**1.1**	**[1.07;1.13]**	** < 0.001**	**1.15**	**0.97–1.52**	**0.012**
PCAT_RCA_	**1.19**	**1.09–1.30**	** < 0.001**	**1.44**	**1.02–2.52**	**0.035**
PCAT_LAD_	**1.7**	**1.33–2.17**	** < 0.001**	**3.10**	**1.19–2.22**	**0.014**
PCAT_LCX_	**2.52**	**1.66–3.83**	** < 0.001**	**2.68**	**1.52–8.91**	** < 0.001**
M	**1.21**	**1.12–1.30**	** < 0.001**	1.17	0.94–1.81	-
V/M	**0.15**	**0.07–0.31**	** < 0.001**	**0.25**	**0.03–0.74**	**0.008**

Abbreviations as in Tables 1 and 2, * Significant difference

**Fig. 3:**
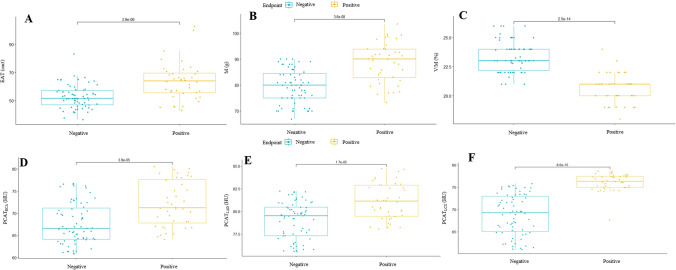
The Boxplot for CCTA-derived parameters in hospital readmission non-ischemic Heart failure. (**A**) CT derived epicardial adipose tissue; (**B**) CT derived cardiac muscle mass; (**C**) CT derived cardiac muscle mass to lumen volume; (**D**) peri-coronary adipose in the right coronary artery; (**E**) peri-coronary adipose in the left anterior descending artery; (**F**) peri-coronary adipose in the circumflex branch of the left coronary artery

Non-ICHF Patients sustaining readmission also had higher EAT (63.9 cm^3^ [-55.5; -69.5] vs. -51.7 cm^3^ [-47.1; -57.2], *P* < 0.01), PCAT_RCA_ (-66.5 HU [-64.1; -71.1] vs. -71.2 HU [-67.8; -77.6], *P* < 0.001), PCAT_LAD_ (-79.0 HU ± 2.0 vs -81.1 ± 2.0, *P* < 0.001), PCAT_LCX_ and CT_M_ (-79.4 HU ± 6.4 vs. -88.6 HU ± 7.5, *P* < 0.001). However, there was no difference between the groups in terms of the CT_FFR_ (0.9 [0.8;0.9] vs. 0.9 [0.8;0.9], *P* = 0.987) (Table 2, Fig. 3).

In univariate logistic regression analysis, the CCTA-derived parameters were associated with readmission among non-ICHF patients (Table 3). The results of multivariate logistic regression indicated that EAT (OR: 1.15, 95% CI: 0.97–1.52, *P* = 0.012), PCAT_RCA_ (OR: 1.44, 95% CI: 1.02–2.52, *P* = 0.035), PCAT_LAD_ (OR: 3.1, 95% CI: 1.19–2.22, *P* = 0.014), PCAT_LCX_ (OR: 2.68, 95% CI: 1.52–8.91, *P* < 0.01) and CT_V/M_ (OR: 0.25, 95% CI: 0.03–0.74, *P* = 0.008).

### Prevalence of variables associated with outcome

**Fig. 4:**
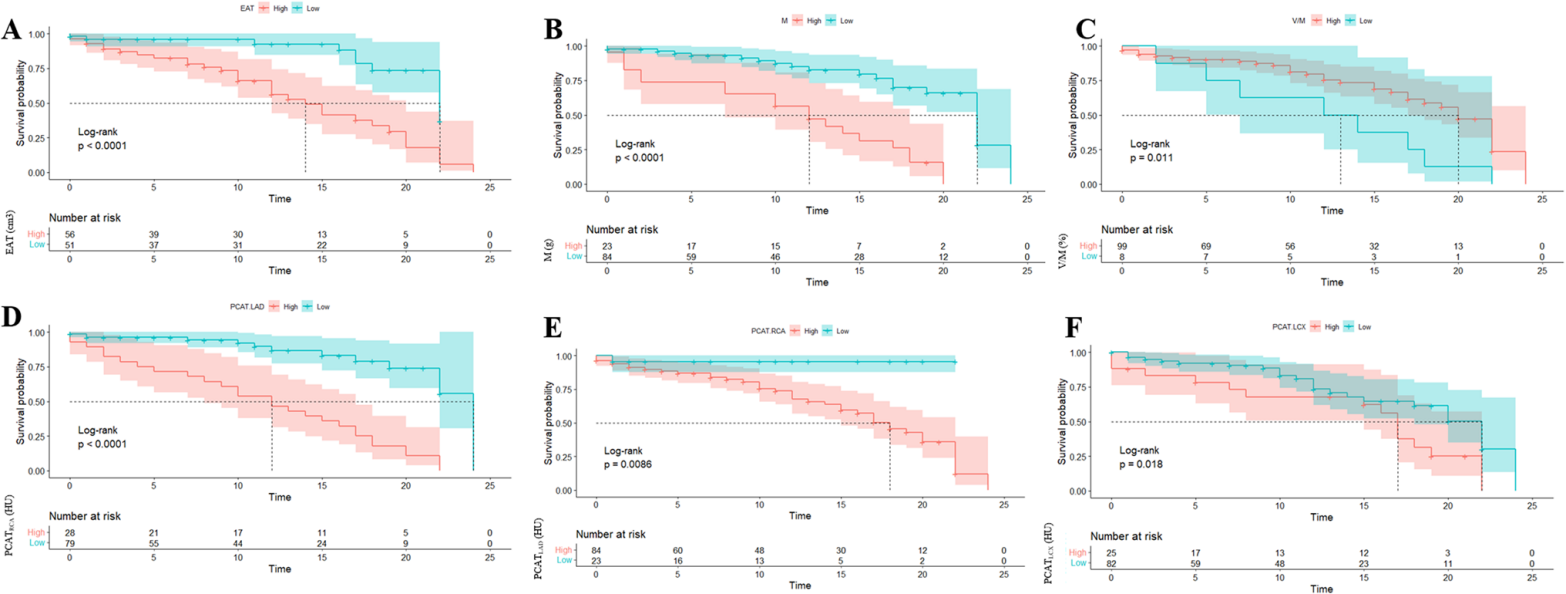
The K-M survival curves of CCTA-derived parameters. (**A**) CTEAT; (**B**) CTM; (**C**) CTV/M; (**D**) PCATLAD; (**E**) PCATRCA; (**F**) PCATLCX

Based on the maxstat, the optimal cut-off values of the EAT and V/M were 54.49 cm^3^ (HR: 1.05; 95% CI: 1.03–1.07; *P* < 0.001) and 20%, respectively, (HR: 0.59; 95% CI: 0.48–0.72; *P* < 0.001) for the endpoint of readmission (Supplementary Fig. 2). Patients with a PCAT_RCA_ lower than -64.68 HU were more likely to be readmitted (HR: 1.1; 95% CI: 1.03–1.16; *P* = 0.002). Patients with PCAT_LAD_ < -81.07 HU (HR: 1.3; 95% CI: 1.1–1.53; *P* = 0.002) or PCAT_LCX_ < -73.42 HU (HR: 1.33; 95% CI: 1.18–1.51; *P* < 0.001) were at the greatest risk of readmission (Table 4, Fig. 4).

**Table 4:** The K-M Analysis for the Prediction of non-ischemic HF readmission

	No event *N* = 67	Event *N* = 40	HR	95%CI	*P*-value
EAT	52.8 (8.21)	63.6 (13.3)	1.05	1.03–1.07	< 0.001*
PCAT_RCA_	-67.7 (4.68)	-72.1 (5.16)	1.1	1.03–1.16	0.002*
PCAT_LAD_	-79.0 (2.02)	-81.1 (1.99)	1.3	1.10–1.53	0.002*
PCAT_LCX_	-68.8 (4.52)	-76.1 (1.96)	1.33	1.18–1.51	< 0.001*
M	79.4 (6.39)	88.6 (7.55)	1.1	1.06–1.15	< 0.001*
V/M	23.4 (1.29)	20.6 (1.25)	0.59	0.48–0.72	< 0.001*
Gander(M)	36 (53.7%)	25 (62.5%)	2.35	1.19–4.61	0.014*
LA	3.73 (0.50)	3.88 (0.45)	2.38	1.21–4.68	0.012*

Abbreviations as in Tables 1 and 2, * Significant difference

**Fig. 5:**
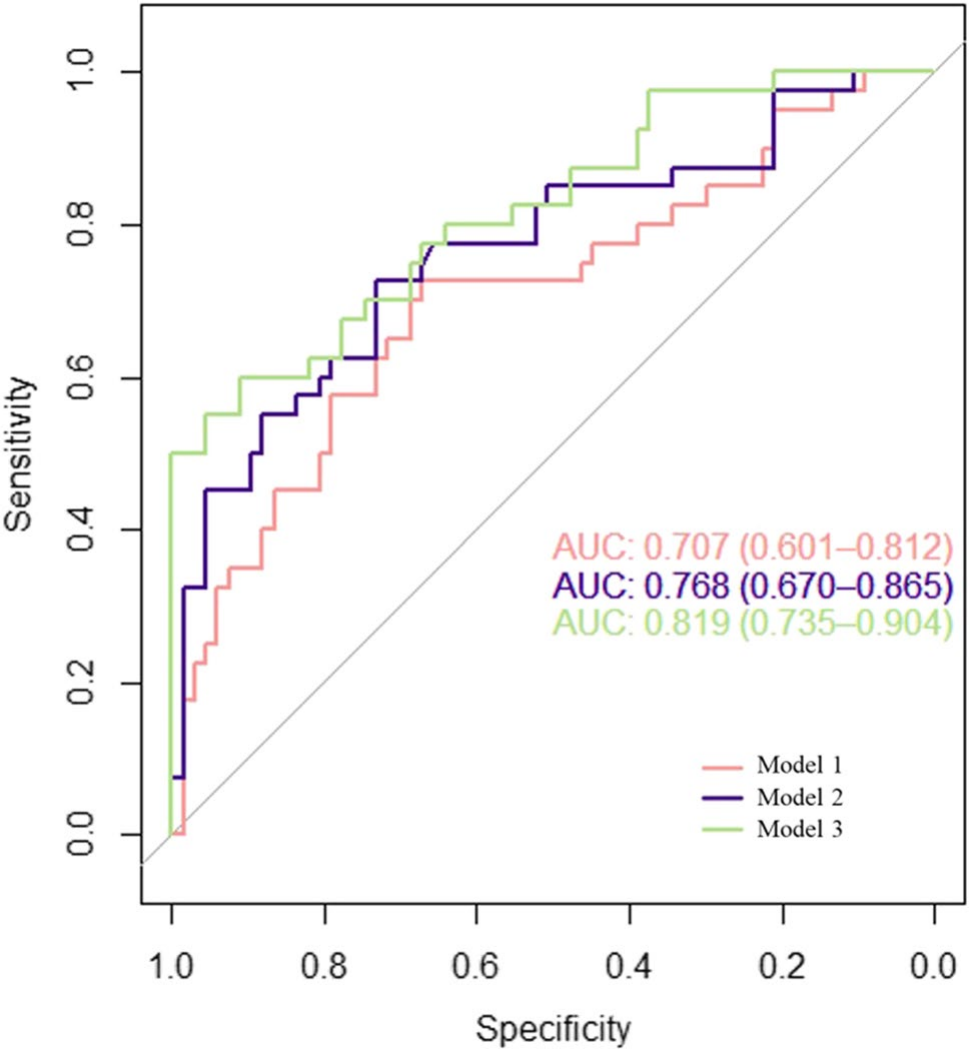
The ROC analysis for the three models

To test whether the CT-derived parameters had predictive value, we compared the AUC curves for 1) the model based on sex and Ao Asc (AUC = 0.71 95% CI: 0.601–0.812); 2) the model based on CT_EAT_, PCAT_RCA,_ PCAT_LAD_, PCAT_LCX_, CT_M_ and CT_V/M_ (AUC = 0.768, 95% CI: 0.67–0.86); and 3) the combined model (AUC = 0.819, 95% CI: 0.735–0.904) (Table 5, Fig. 5). The CCTA-derived volume was not associated with an increased risk of readmission.

**Table 5:** Results of ROC calculations for the three model

	AUC	SEN	SPE	PLR	NLR	Youden index	PPV	NPV	DOR
Model 1	0.71	0.73	0.67	2.21	0.41	0.4	0.57	0.8	5.4
Model 2	0.77	0.73	0.73	2.70	0.38	0.46	0.62	0.82	7.14
Model 3	0.82	0.60	0.91	6.70	0.44	0.51	0.8	0.79	15.2

AUC Area Under Curve, SEN sensitivity, SPE Specificity, PLR positive likelihood ratio, NLR negative likelihood ratio, PPV positive predictive value, NPV negative predictive value, DOR diagnostic odds

## Discussion

Several novel and clinically important findings were identified in our study. First, higher epicardial fat and pericoronary adipose tissue were observed in non-ICHF patients than in healthy people and are related to readmission. Second, the coronary lumen volume to cardiac muscle mass ratio (V/M) and left ventricular myocardial mass (M) were independently associated with poor prognosis. To our knowledge, this is the first reporting of the associations between readmission and risk factors, including CT_EAT_, CT_V/M_, CT_FFR_ and CT_PCAT,_ for non-ICHF.

Obesity is prevalent among HF patients, but whether it can serve as an independent prognostic factor for HF outcomes remains a topic of debate [21]. Some relevant studies have suggested that overweight and mildly to moderately obese HF patients may have longer survival rates than normal-weight or underweight HF patients [22, 23]. The potential explanation for this difference may be related to whether fat leads to excessive accumulation of WAT and myocardial fat infiltration [24, 25]. We hypothesized that, compared to BMI, EAT might be a more precise indicator of obesity, and thus, measuring EAT could better assess disease progression. In fact, previous research has confirmed that EAT participates in the occurrence and progression of cardiovascular diseases by synthesizing and secreting proinflammatory mediators and neurohormones such as IL-1β, IL-6, TNF-α, MCP-1, resistin, visfatin, and others [23]. Currently, research on EAT in HF patients is primarily focused on those classified based on ejection fraction (EF) [26]. Studies by Van Woerden and Obokata, utilizing cardiac magnetic resonance (CMR) and echocardiography, found that EAT mass and thickness were higher in HF with preserved EF (HFpEF) patients than in normal individuals [27, 28]. Liu et al. discovered that measuring EAT volume and density via CT scans could better predict the prognosis of patients with HFpEF [29]. Therefore, our research findings, based on CCTA, reveal that EAT levels in patients with non-ICHF often exceed those in individuals with normal cardiac function, consistent with the aforementioned research results.

In our study, we observed that patients with elevated amounts of EAT often exhibited an increased risk of readmission. The potential explanation for this observation may be linked to the detrimental impact of EAT on cardiac structure and function through endocrine and vascular pathways. Research indicates that the accumulation of EAT can lead to increased myocardial wall thickness, affecting cardiac diastolic function [30]. Excessive fat accumulation in EAT may also promote inflammation by releasing proinflammatory cytokines (such as adiponectin) into adjacent myocardium, thereby fostering microvascular inflammation and fibrosis, ultimately leading to atrial and ventricular functional impairment and, consequently, myocardial dysfunction [31].

The results of the NeXtsTeps (NXT) trial support the hypothesis of vascular involvement and microcirculatory dysfunction in HF patients [32]. However, in clinical practice, the assessment of microcirculatory dysfunction following myocardial infarction is often conducted using CMR or CT myocardial perfusion imaging, which requires advanced equipment and significant patient cooperation, making it challenging for widespread implementation [6]. In this study, we employed the ratio of coronary artery luminal volume to left ventricular mass, which reflects cardiac metabolic capacity without the need for additional pharmacological agents. Among patients with microvascular angina (MVA), V/M, as an indicator of myocardial oxygen demand, was significantly lower than in healthy individuals, consistent with the findings of this study [33]. We hypothesize that in readmitted non-ICHF patients, the microvessels undergo endothelium-dependent vasodilatory injury, thereby impacting myocardial contractility and relaxation. Calculating CT_V/M_ can further enhance our understanding of the pathophysiology of cardiovascular diseases.

Inflammation has been shown to be linked to the development, progression, and worsening prognosis of HF [33, 34]. Assessment of pericoronary adipose tissue is a validated technique that allows the direct detection of coronary inflammation on CCTA. Proinflammatory cytokines released by the coronary arteries in the presence of inflammation inhibit adipocyte maturation, leading to smaller adipocytes and more water content [18]. In the present study, we found for the first time a reduction in the pericoronary fat attenuation coefficient, especially in the RCA, in readmitted patients with non-ICHF. Interestingly, the CRISP-CT study also demonstrated that the fat attenuation index (FAI) around the RCA can effectively predict myocardial ischaemia and the risk of high-risk plaques in coronary artery disease patients [33, 35].

Hence, our research outcomes are in concurrence with the predominant body of studies, demonstrating that elevated amounts of PCATs are linked to elevated risk, particularly in the context of the RCA. This phenomenon may be due to the accumulation of adipose tissue surrounding the coronary arteries, resulting in microvascular circulatory blockage, which influences the prognostic outlook of individuals suffering from non-ICHF.

In conclusion, CT_EAT_, CT_PCAT_, and CT_V/M_ are associated with the prognosis of non-ICHF patients. Therefore, based on the above parameters, CCTA provides a multilevel and noninvasive evaluation option for non-ICHF patients that combines information on coronary arteries, cardiac structure and function, cardiac fat and other aspects and is expected to become a powerful tool for prognostic assessment and treatment decision-making, providing new hope for improving the management and prognosis of non-ICHF patients.

### Limitations

First, this was a retrospective study conducted at a single centre, with relatively small sample sizes in each cohort, constraining the data we could analyse, such as waist circumference, waist-to-hip ratio, and educational level. Second, we focused solely on the presence of non-ICHF and did not perform subgroup analyses based on EF among HF patients. Third, due to radiation dose considerations in this study, we opted for prospectively collected CCTA data, limiting the assessment to EAT, PCAT, and V/M measurements. Comprehensive cardiac functional analysis based on the entire cardiac cycle was not feasible. Fourth, regarding the determination of optimal cut-off values, we chose to utilize maxstat for the calculations [20]. Compared to ROC curves, the maxstat method is typically less influenced by subjective judgements since it relies on the inherent statistical properties of the data, obviating the need for researcher subjectivity.

### Future directions

In the future, a well-designed prospective study incorporating a larger number of cases and patient data will be essential to corroborate our findings. Additionally, long-term follow-up data can help enhance and refine model construction.

### Supplementary Information

Below is the link to the electronic supplementary material.

Supplementary file1 (DOCX 197 KB)

## Data Availability

The data provided include raw data, processed data, analytical tools used, which support the findings of this study. For further inquiries about the datasets, please contact the corresponding author.

## Abbreviations

BMI: Body mass index
CHO: Cholesterol
TG: Triglyceride
HDL-C: High-density lipoprotein cholesterol
LDL-C: Low-density lipoprotein cholesterol
ApoA1: Apolipoprotein A1
ApoB: Apolipoprotein B
FFA: Free fatty acid
BNP: B-type natriuretic peptide
NT-proBNP: Pro-B type natriuretic peptide
CT_EAT_: CT-derived epicardial adipose tissue
CT_PCAT_: CT-derived pericoronary adipose tissue
CT_V/M_: CT-derived cardiac lumen volume to muscle mass
CT_FFR_: CT-derived fractional flow reserve
ICA: Invasive coronary angiography
EF: Ejection fraction
IVSd: Interventricular septal thickness at diastole
LVIDd: Left ventricular internal diameter at end-diastole
LVPWd: Left ventricular posterior wall dimensions
IVSs: Left ventricular systolic diameter
LVIDs: Left ventricular systolic diameter
LVPWs: Left ventricular posterior wall thickness
AO-stj: Aortic arch diameter
LA-ap: Left atrial diameter
