# Supplementary material for: The Role of CCTA-derived Cardiac Structure and Function Analysis in the Prediction of Readmission in Nonischemic Heart Failure
Source: J Cardiovasc Transl Res. 2024 Jan 26;17(1):216–26. doi: 10.1007/s12265-023-10467-6 (PMC10896797; doi:10.1007/s12265-023-10467-6)
Supplement: Supplementary file 1 — Supplementary file1 (DOCX 197 KB) [file 12265_2023_10467_MOESM1_ESM.docx]

Supplementary Material

The role of CCTA-derived cardiac structure and function analysis in the prediction of readmission in nonischemic heart failure

# Chengjia Liu1, shuangxiang Lin1, Yangyang Sheng1, Xinghong Wang1, Jianzhong Sun1, Wu Jiaxing2, Risheng Yu1

# 1 Department of Radiology, The Second Affiliated Hospital Zhejiang University School of Medicine, Hangzhou, Zhejiang, China

# 2 Siemens Healthineers, No.399, West Haiyang Road, Shanghai, 200126, ChinaSupplementary Figures

## Supplementary Figures

**
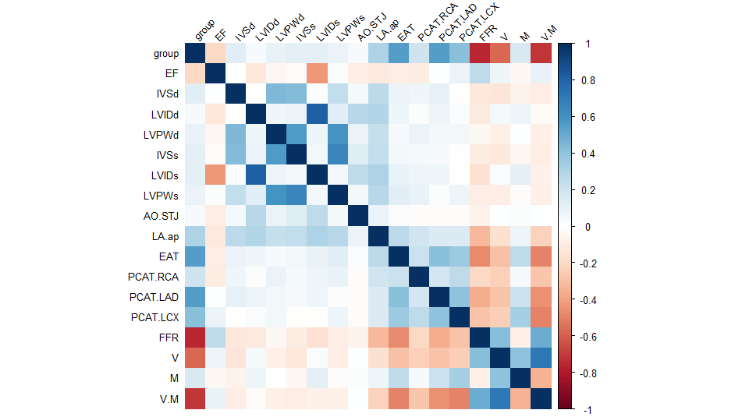
**

**Supplementary Figure 1.** The correlations between Healthy control and Non-ischemic HF.

**
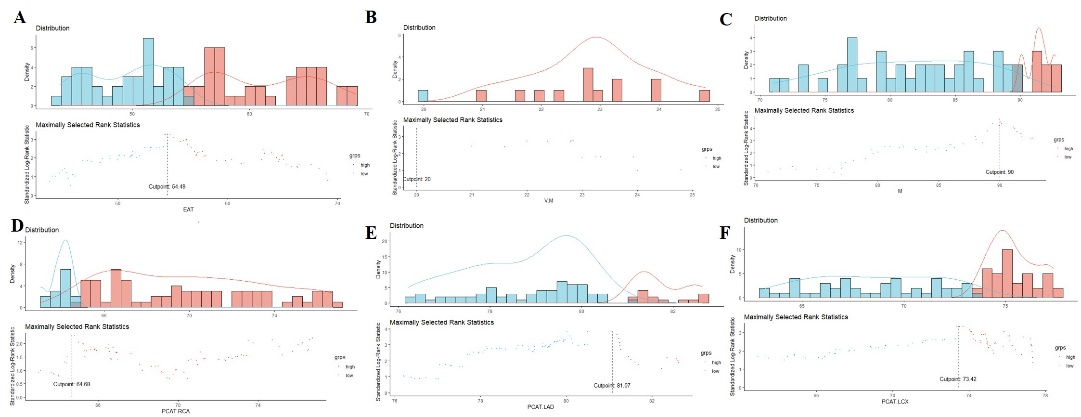
**

**Supplementary Figure 2.** The he maximally selected rank statistics(maxstat) further visualizations and examples of CCTA-derived parameters. (A) CT_EAT_;(B) CT_V/M_;(C) CT_M_;(D) PCAT_LAD; (E) PCAT_LAD;(F) PCAT_LCX;
